# Weak-keys and key-recovery attack for $\text{ TinyJAMBU }$

**DOI:** 10.1038/s41598-022-19046-2

**Published:** 2022-09-29

**Authors:** Pranjal Dutta, Mahesh Sreekumar Rajasree, Santanu Sarkar

**Affiliations:** 1 Department of Computer Science, Chennai Mathematical Institute, Chennai, India; 2 Department of Computer Science, Indian Institute of Technology Kanpur, Kanpur, India; 3 Department of Mathematics, Indian Institute of Technology Madras, Chennai, India

**Keywords:** Computer science, Information technology

## Abstract

In this paper, we study NIST lightweight 3*rd* round candidate $\text{ TinyJAMBU }$. The core component of $\text{ TinyJAMBU }$ is the keyed permutation $\mathcal {P}_n$, which is based on a non-linear feedback shift register. By analysing this permutation carefully, we are able to find *good* cubes that are used to build distinguishers in the *weak-key* setting. In particular, we show that there are at least $2^{108}$ keys for which TinyJAMBU can be distinguished from a random source for up to 476 rounds. These distinguishers outperform the best-known distinguishers, which were proposed in ‘Scientific Reports - Nature’ by Teng et al. We are the *first* to study the *exact* degree of the feedback polynomial $\mathcal {P}_n$ in the nonce variables. This helped us in concluding that $\text{ TinyJAMBU }$ with more than 445 rounds is secure against distinguishers using 32 sized cubes in the normal setting. Finally, we give new key-recovery attacks against $\text{ TinyJAMBU }$ using the concepts of monomial trail presented by Hu et al. at ASIACRYPT 2020. Our attacks are *unlikely* to jeopardise the security of the entire 640 rounds $\text{ TinyJAMBU }$, but we strongly anticipate that they will shed new lights on the cipher’s security.

## Introduction

Numerous well-known cryptographic algorithms have been constructed using Nonlinear Feedback Shift Registers (NFSRs), including stream cipher Trivium^1^, authenticated cipher ACORN^2^, block cipher KATAN^3^, and hash function Quark^4^. Cryptosystems based on NFSRs can be represented using tweakable Boolean functions that contain both key and IV variables. A cryptographic primitive with a low algebraic degree is susceptible to a wide variety of well-known attacks, including higher order differential attacks^5,6^, algebraic attacks^7,8^, cube attacks^9^, and integral attacks^10^. But because the algebraic degree is so high, it is hard to figure out the exact value of the algebraic degree for cryptographic primitives. Interestingly, a number of theoretical tools^11–13^ have been developed for estimating the upper bound on the algebraic degree of iterated permutations.

The most precise and general technique for identifying integral distinguishers is the *division property*^13^. The propagation of the division property was initially investigated using the breadth-first search technique, but it is computationally problematic for ciphers with high block sizes. Then, Xiang et al.^14^ introduced an MILP-based algorithm based on the beneficial notion of the division trail. In Eurocrypt 2009, Dinur and Shamir^9^ proposed the cube attack. There is a concept known as *Superpoly* in the cube attack. Superpolies may be used to recover the cipher’s secret key. Previously, superpolies could only be retrieved in an experimental manner. Later, the division property was added to the cube attacks for the first time in^15^, and it allows us to determine the secret variables.

One of the most significant security criteria for a keyed cryptographic primitive is its unpredictable behaviour with regard to any randomly chosen key from the whole key space. When a key is used with a given cipher, it is considered to be *weak* if it causes the cipher to behave in an undesirable way (like if it reduces the algebraic degree significantly). The goal of a cipher design is to have a flat keyspace i.e., all keys should be equally strong. DES is probably the most well-known example of weak-keys which features a few specific keys that are referred to as *weak-keys*^16^. Apart from DES, many attacks in the weak-key setting for block cipher^17–19^ as well as stream ciphers^20,21^ have been presented. In the weak-key setting, the time complexity of a distinguisher or a key-recovery attack should be less than the number of weak-keys. However, finding a weak-key set is a computationally hard problem. The *invariant subspace attack*^22,23^, for example, is a decent general weak-key attack, that is known in the literature. Recently, cube attacks that investigate key conditions which may lead to weak-key attacks, have been proposed in^24–26^. Some interesting applications of cryptology have been proposed in^27–30^.

NIST^31^ has launched a process for soliciting, evaluating, and standardising lightweight cryptographic algorithms suited for use in limited contexts when the performance of current NIST cryptographic standards is unacceptably low. In August 2018, NIST issued a call for algorithms to be considered for lightweight cryptography standards. There were initially 57 submissions. NIST picked 32 candidates in the second round of trimming on August 30 2019. On March 29, 2021, NIST released ten candidates following the third round of pruning. $\text{ TinyJAMBU }$ is one of these candidates. $\text{ TinyJAMBU }$ employs a keyed-permutation based on an NLFSR that computes only a single NAND gate as a non-linear component per round. It is a small variant of the JAMBU mode, which is the smallest block cipher authenticated encryption mode in the CAESAR competition^32^, and it was selected to the third round of the competition.

**Prior works.** In^33,34^, the designers of $\text{ TinyJAMBU }$ analysed the system against various attacks. They analysed the differential properties of keyed permutation $\mathcal {P}_n$ which is the core component of $\text{ TinyJAMBU }$. They demonstrated that the differential probability for $\mathcal {P}_{640}$ rounds is *insignificant*. They also studied the linear properties of $\mathcal {P}_n$ claiming that 32 sized cube attacks are ineffective against $\mathcal {P}_n$, when $n \ge 512$.

Using Mixed Integer Linear Programming (MILP), the designers^33^ count the least number of active AND gates to find differential and linear trails. This counting technique, however, disregards the inter-dependency between several AND gates. This flaw was identified by Saha et al.^35^. While the designers suggested the 384-round differential trail with probability $2^{-80}$ by regarding each AND gate independent,^35^ confirmed that there is no such trail by taking into account the dependency. Further, they proposed a forgery attack with complexity $2^{62.68}$ on 338 rounds and a differential trail with probability $2^{-70.68}$ for 384 rounds using their refined model.

Recently, Teng et al.^36^ looked into the TinyJAMBU cipher’s resistance to cube attacks. They showed key-recovery attack for 428 rounds and distinguishing attack for 438 rounds using small size cubes.

### Our contributions

The major focus of this paper is to study $\text{ TinyJAMBU }$ from three important and different contexts – (i) the *weak-key* setting, (ii) understanding the *exact* degree of the feedback polynomial in the nonce variables (iii) the *key-recovery* attacks. Most significantly, this is the *first* time that the concept of *monomial trail*, introduced in^37^, and the MILP tool have been employed together in analyzing $\text{ TinyJAMBU }$.

1. We begin by studying $\text{ TinyJAMBU }$ in the *weak-key* setting. Since the core component of $\text{ TinyJAMBU }$ is the keyed permutation $\mathcal {P}_n$, we analyse the structure of $\mathcal {P}_n$ for any weakness. Using this, we present a class of *good* cubes which will help us in our attack. We show that there are at least $2^{108}$ keys for which $\text{ TinyJAMBU }$ can be distinguished from a random source for up to 476 rounds.
2. Next, we focus on determining the *exact* degree in the nonce variables of the feedback polynomial. Using the monomial trail concepts^37^ and MILP tool, we demonstrate that after 381 rounds, the degree equals 32. This is the *first* time one has studied the exact degree of the feedback polynomial of $\text{ TinyJAMBU }$. Moreover, the exact degree from our experiments implies that $\text{ TinyJAMBU }$ is secure against cube attacks with 32-dimension cubes after 445 rounds.
3. Finally, we present key-recovery attacks for reduced round $\text{ TinyJAMBU }$. We again use monomial trail concept and MILP tool to find superpolies consisting of key variables only for 440 rounds. This leads to an *improved* key-recovery attack, which is better than the 428 round key-recovery attack presented by Teng et. al.^36^, in their recent result published in Scientific Reports - Nature.

**Roadmap of the Paper.** The rest of the paper is organized as follows. In “Preliminaries and notations” section, we first describe the specification of $\text{ TinyJAMBU }$. Following that, we go through some relevant cryptanalytic techniques. In “[Sec Sec10]” section, we discuss practical weak-key distinguishers for round-reduced $\text{ TinyJAMBU }$. The procedure for determining the exact degree of the feedback polynomial has been presented in “[Sec Sec14]” section. “Key-recovery attacks” section discusses key-recovery attacks for reduced round $\text{ TinyJAMBU }$. Finally, we conclude in “Conclusion” section with future research directions.

## Preliminaries and notations

### Notations

We will use bold font to denote vectors and sets and normal font to denote the component of a vector/set.

1. Fi For any vector $\textbf {u}$ of dimension *n*, we denote the *i* *th* coordinate of $\textbf {u}$ as $u_i$, therefore, $\textbf {u}$ can be written as $[u_1, u_2, \dots , u_n]$.
2. Fi For a Boolean vector $\textbf {u}\in \{0,1\}^n$, we define the weight of $\textbf {u}$ as $\mathsf {wt}(\textbf {u}) = \sum _{i=1}^n u_i$.

**Polynomials.** Let $f(\textbf {x}) \in \mathbb {F}_2[\textbf {x}]$ be a Boolean polynomial over *n*-variables $x_1, \dots , x_n$. Then, the algebraic normal form of $f(\textbf {x})$ is

1 $$ f({\mathbf{x}}) = \mathop  \oplus \limits_{{{\mathbf{u}} \in 0,1^{n} }} \alpha _{{\mathbf{u}}} \pi _{{\mathbf{u}}} ({\mathbf{x}}) $$

where $$\alpha _{\textbf {u}} \in \mathbb {F}_2$$ and

2 $$\begin{aligned} \pi _{\textbf {u}}(\textbf {x}) = \prod _{i = 1}^n x_i^{u_i} \end{aligned}$$

We will use the notation $\pi _{\textbf {u}}(\textbf {x}) \rightarrow f(\textbf {x})$ when $\pi _{\textbf {u}}(\textbf {x})$ is a monomial in $f(\textbf {x})$, i.e., $\alpha _{\textbf {u}} = 1$. For any index set $I \subseteq n$, we will use $\textbf {x}_I$ to denote $\underset{i \in I}{\prod } x_i$ and $\textbf {x}_{\overline{I}}$ to denote $\underset{i \in [n] \setminus I}{\prod } x_i$.

### Description of $\text{ TinyJAMBU }$

Wu and Huang designed $\text{ TinyJAMBU }$ ^33,34^ which is a variant of JAMBU^38^. It is a family of lightweight authenticated encryption algorithms and one among the 10 finalists in the NIST Lightweight Cryptography (LWC) Standardization project^31^. The family consists of three variants - $\text{ TinyJAMBU }$-128, $\text{ TinyJAMBU }$-192 and $\text{ TinyJAMBU }$-256 as shown in Table 1.

**Table 1:** $\text{ TinyJAMBU }$ variants and their recommended parameters.

Name	State size	Size of			Rounds	
		Key	Nonce	Tag	$\mathcal {Q}$	$\hat{\mathcal {Q}}$
$\text{ TinyJAMBU }$-128	128	128	96	64	640	1024
$\text{ TinyJAMBU }$-192	128	192	96	64	640	1152
$\text{ TinyJAMBU }$-256	128	256	96	64	640	1280

The core permutation $\mathcal {P}_n$ updates the state using a non-linear feedback shift register for *n*-rounds where the *i* *th* rounds is described in Algorithm 1. We will use the notation $\mathcal {P}$ for $\mathcal {P}_1$. The permutations $\mathcal {Q}$ and $\hat{\mathcal {Q}}$ mentioned in Table 1 are $\mathcal {P}_n$ with different values of *n*.
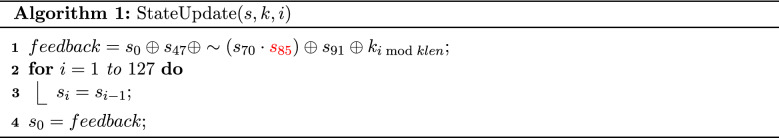


### Specification of $\text{ TinyJAMBU }$-128

As shown in Table 1, $\text{ TinyJAMBU }$-128 has a 128-bit state and key whereas the number of nonce bits is 96. The tag size is 64 bits. The permutation function ${\mathcal {Q}}$ is $\mathcal {P}_{640}$ whereas ${\hat{\mathcal {Q}}}$ is $\mathcal {P}_{1024}$.

**Figure 1:**
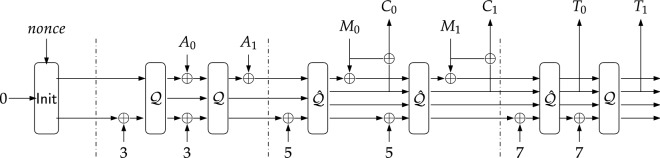
$\text{ TinyJAMBU }$’s mode of operation (encryption phase).

The authenticated encryption algorithm of $\text{ TinyJAMBU }$ can be divided into four phases as shown in Fig. 1: Initialisation, associated data processing, encryption and finalisation.

**Initialization:** In this phase, the state is set to all zero and is updated by the keyed permutation $\hat{\mathcal {Q}}$. The 96 bit nonce is divided into 3 parts- $nonce_0, nonce_1$ and $nonce_2$ of equal size and updates the state using Algorithm 2.
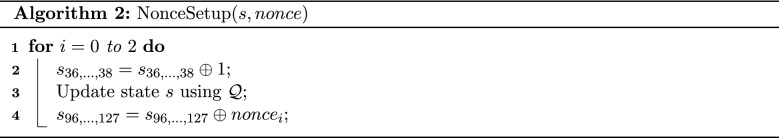


**Associated date processing:** After the initialization phase, the associated date $(A_0, A_1)$ are processed by $\mathsf {XOR}$ing them to the state and updating it using the keyed permutation $\mathcal {Q}$ as described in Algorithm 3.
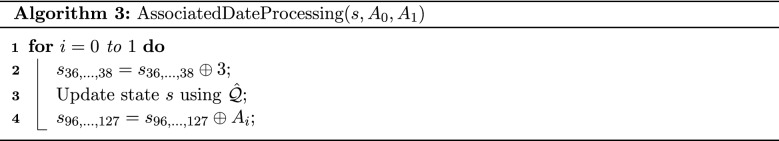


**Encryption:** In the encryption phase, the message *M* is encrypted to produce the ciphertext *C*. In the paper, we will always assume that the size of the message *M* denoted by $\ell$ is a multiple of 32. In Fig. 1, we have assumed that *M* consists of 64 bits. In general, Algorithm 4 is performed $\ell /32$ times on $M_0, \dots , M_{\ell /32 - 1}$ where $M_i$ is the *i* *th* block of *M* having size equal to 32.
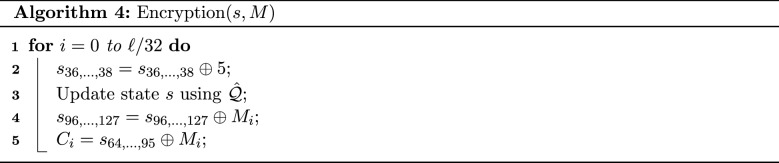


**Tag generation:** Finally, the 64-bit tag $T = (T_0, T_1)$ is generated as shown in Algorithm 5.
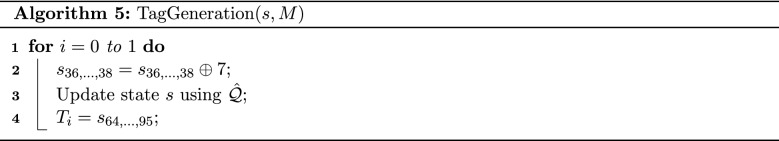


### Cube attack

Here we provide a high-level overview of the cube attack model. To initialise its state, a stream cipher typically uses one secret key, $\textbf {k}$, and a set of public variables $\textbf {x}$. The secret key remains secret, and it is known only to the encryptor and the decryptor.

However, the $\textbf {x}$ (or nonce) is considered a public variable. Keystream bits are typically generated by the ciphers after some initialization rounds. By interpreting the keystream bit *z* as a Boolean polynomial over the secret keys $\textbf {k}$ and $\textbf {x}$, it can be expressed as $z = f(\textbf {x},\textbf {k})$. Let secret key variable $\textbf {k}=(k_1,\ldots ,k_{l})$ and public variable $\textbf {x}=(x_1,\ldots ,x_{m})$. The cube attack is based on the principle of simplifying the polynomial $z = f(\textbf {x}, \textbf {k})$ in order to obtain the value of $\textbf {k}$. Let us set the cube indices $\textbf {I}= \{i_1, \ldots , i_c\} \subseteq \{1,2, \ldots , m\}$ in the preprocessing phase. Then we can express $f(\textbf {x}, \textbf {k})$ as

3 $$\begin{aligned} f(\textbf {x},\textbf {k})=x_{i_1} \cdots x_{i_c} p_{\textbf {I}}(\textbf {x},\textbf {k}) + q_{\textbf {I}}(\textbf {x}, \textbf {k}) \end{aligned}$$

where each monomial of the function $q$ misses at least one variable from $\{x_{i_1}, x_{i_2}, \cdots x_{i_c}\}$.

In order to use the attack during the online phase, we only need to know its superpoly $p_{\textbf {I}}$, and we can set the values of non-cube variables $x_i$ to 0 (or to 1) to simplify the long superpoly expression. Consider the $C_{\textbf {I}}=\{(x_{i_1},\ldots ,x_{i_c}): x_j\in \{0,1\} ~\text{ for }~j\in \{i_1,\ldots ,i_c \} \}$. The superpoly can then be recovered because $\sum _{(x_{i_1},\ldots ,x_{i_c})\in C_I} f(x_1,\ldots ,x_{m},k_1,\ldots ,k_{l}) =p_{\textbf {I}}$.

Thus, the attacker first finds some cube variables and then computes the sum of the output bits for all possible values of the cube variables to determine the value of the superpoly. The attacker’s objective is to select cube variables and fix the remaining public variables in such a way so that the superpoly is reduced to a linear function in secret variables.

The main point of concern is that after a large number of initialization rounds, the expression of the output bit always becomes very much complicated.

In reality, after a few rounds of initialization, it is not possible to compute the algebraic expression of the output bit in the latest stream ciphers because of the way they are designed. To tackle this problem, the attacker uses the cipher as a blackbox. He randomly chooses the cube variables, and the blackbox provides the output bits corresponding to all possible values of the cube variables. The value of the superpoly can be recovered by the attacker by performing the sum on the values that have been retrieved. The BLR linearity test^39^ can be used by the attacker to determine whether or not the superpoly is linear. A more in-depth description of the cube attack may be found in^9^.

In 2009, Aumasson et al.^40^ proposed cube tester to assess the non-randomness of a Boolean function. The presence of a monomial, balancedness, constantness, presence of linear variables, and the presence of neutral variables can be determined by using cube tester. A Boolean function is said to be vulnerable if it can be distinguished by some property such as the ones mentioned above.

### Upper bound of degree

Given a Boolean polynomial *f* in *n* variable, we want to find its algebraic degree after fixing some variables. Let $x_1,\ldots ,x_{n}$ be the variables of the function *f*.

Assume that $x_1,\ldots , x_{k}$ are initially fixed to 0. Now we are interested to check whether the reduced polynomial (after setting $x_1,\ldots ,x_{k}$ to 0) has degree $n-k$ or not. For this purpose, we do the following:

1. Consider $x_{k+1},x_{k+2},\ldots ,x_{n}$ as cube variables.
2. Calculate the cube sum on the reduced polynomial over the prescribed cube variables.
3. If the cube sum is zero, then we conclude that the degree of the reduced polynomial is strictly less than $n-k$.

### Monomial trails

#### **Definition 1**

(*Monomial trail*) Let $\textbf {x}^{(i+1)} = f^{(i)}(\textbf {x}^{i})$ for $0 \le i < r$. We say a monomial trail exists from $\pi _{\textbf {u}^{(0)}}(\textbf {x}^{(0)})$ to $\pi _{\textbf {u}^{(r)}}(\textbf {x}^{(r)}) = \pi _{\textbf {u}^{(r)}}(f^{(r-1)}(\textbf {x}^{(r-1)}))$ (denoted as $\pi _{\textbf {u}^{(0)}}(\textbf {x}^{(0)})  {\leadsto }\pi _{\textbf {u}^{(r)}}(\textbf {x}^{(r)})$) if there exists a sequence of monomials $(\pi _{\textbf {u}^{(0)}}(\textbf {x}^{(0)}), \pi _{\textbf {u}^{(1)}}(\textbf {x}^{(1)}), \dots , \pi _{\textbf {u}^{(r)}}(\textbf {x}^{(r)}))$ such that $\pi _{\textbf {u}^{(i)}}(\textbf {x}^{(i)}) \rightarrow \pi _{\textbf {u}^{(i+1)}}(\textbf {x}^{(i+1)})$ for all $0 \le i < r$.

It is important to note that there can be multiple monomial trails from $\pi _{\textbf {u}^{(0)}}(\textbf {x}^{(0)})$ to $\pi _{\textbf {u}^{(r)}}(\textbf {x}^{(r)})$. Observe that the existence of a monomial trail from $\pi _{\textbf {u}^{(0)}}(\textbf {x}^{(0)})$ to $\pi _{\textbf {u}^{(r)}}(\textbf {x}^{(r)})$ does not necessarily imply that $\pi _{\textbf {u}^{(0)}}(\textbf {x}^{(0)})$ is a monomial in $\pi _{\textbf {u}^{(r)}}(\textbf {x}^{(r)})$. We can only guarantee the converse, i.e.,

#### Claim 1

If $${\pi} _{{\textbf {u}}^{(0)}}({\textbf {x}}^{(0)}) \not  \leadsto {\pi} _{{\textbf {u}}^{(r)}}({\textbf {x}}^{(r)})$$ then $${\pi} _{{\textbf {u}}^{(0)}}({\textbf {x}}^{(0)}) \not \rightarrow {\pi} _{{\textbf {u}}^{(r)}}({\textbf {x}}^{(r)})$$.

Let us denote $|\pi _{\textbf {u}^{(0)}}(\textbf {x}^{(0)})  {\leadsto }\pi _{\textbf {u}^{(r)}}(\textbf {x}^{(r)})|$ the number of distinct monomial trails from $\pi _{\textbf {u}^{(0)}}(\textbf {x}^{(0)})$ to $\pi _{\textbf {u}^{(r)}}(\textbf {x}^{(r)})$. Then, the monomial $\pi _{\textbf {u}^{(0)}}(\textbf {x}^{(0)})$ exists in $\pi _{\textbf {u}^{(r)}}(\textbf {x}^{(r)})$ if the number is odd.

#### Claim 2

If $|\pi _{\textbf {u}^{(0)}}(\textbf {x}^{(0)})  {\leadsto }\pi _{\textbf {u}^{(r)}}(\textbf {x}^{(r)})|$ is odd, then $\pi _{\textbf {u}^{(0)}}(\textbf {x}^{(0)}) \rightarrow \pi _{\textbf {u}^{(r)}}(\textbf {x}^{(r)})$.

Therefore, Claim 2 gives us a procedure to find whether a monomial exists in a polynomial or not. For more details on monomail trails, we refer to^37^.

## Weak-keys for $\text{ TinyJAMBU }$

In this section, we will present a new cube distinguisher for 451 rounds $\text{ TinyJAMBU }$ that works for $2^{101}$ keys. We also show another distinguisher for 476 rounds for $2^{80}$ keys. We will first analyse the feedback polynomial in the permutation $\mathcal {P}$ which will help to find *good* cubes. Using these cubes, we will further study the key variables involved in the feedback polynomials, which will help us to find weak-keys for $\text{ TinyJAMBU }$.

To be more precise, let us consider the scenario of a traditional cube attack as a distinguisher. Let $\textbf {x}$ be the set of all public (nonce) variables, $\textbf {k}$ be the set of key variables and $f(\textbf {x},\textbf {k})$ be the output polynomial. Then, for any cube $\mathcal {C}_{\textbf {I}}$ where $\textbf {I}\subseteq [n]$ is the index set and *n* is the number of public variables, we have

4 $$\begin{aligned} f(\textbf {x},\textbf {k}) = \left( \prod _{i \in \textbf {I}} x_i \cdot p_{\textbf {I}}(\textbf {x},\textbf {k}) \right) + q_{\textbf {I}}(\textbf {x},\textbf {k}), \end{aligned}$$

where $p_{\textbf {I}}(\textbf {x},\textbf {k})$ *does not* contain any variable $x_i$, where $i \in \textbf {I}$ and every monomial of $q_{\textbf {I}}(\textbf {x}, \textbf {k})$ is not divisible by $\prod _{i \in \textbf {I}} x_i$. In a cube attack, one takes advantage of the fact that for a properly chosen cube $\mathcal {C}_{\textbf {I}}$ and constant values $a_i, \forall i \in [n] \setminus \textbf {I}$, the superpoly of the output polynomial $f(\textbf {x},\textbf {k})$ is a *constant*, i.e., $p_{\textbf {I}}(\textbf {A}, \textbf {k})$ is a constant, where $\textbf {A}= \{a_i \ |\ i \in I\}$. Observe that for $p_{\textbf {I}}( \textbf {A}, \textbf {k})$ be to a constant, its degree with respect to $\textbf {k}$ must be 0.

Therefore, the above attack fails when there is no possible cube with appropriate constant values that has a superpoly equal to a constant. This happens when the number of rounds increases because the degree of the polynomials with respect to both public and key variables increases exponentially. Nevertheless, if we can find the superpoly $p_{\textbf {I}}(\textbf {A}, \textbf {k})$ which is a polynomial in the key variabes only, then we can use this information to find *weak-keys* such that $p_{\textbf {I}}(\textbf {A},\textbf {k})$ is a constant. These weak-keys are essentially ones that satisfies the equation $p_{\textbf {I}}(\textbf {A},\textbf {k}) = constant$.

But, to find out the superpolies is itself a difficult task. Using MILP, one can find superpoly. However this technique is only possible for small rounds because the degree of the superpolies will be small. Therefore, instead of extracting the exact superpolies, we will show a method to decrease the degree of the superpoly by imposing some constraints on the key variables. By doing so, we will get weak-keys such that the superpoly is a constant with some probability.

### Weak-keys attacks using cubes from^36^

In^36^, the authors presented several cube attacks for $\text{ TinyJAMBU }$. In their experiments, the authors devised five different cube attacks, DA1 to DA5, depending on the scenario. DA1 and DA2 are attacks against the initialization phase, i.e.,

- DA1 uses only first 64 bits of nonce (i.e., $nonce_0$ and $nonce_1$ only) and can observe the keystream after the initialization phase. But, the total number of permutation rounds being considered is $1024 + 3 \times 384$. This is according to^33^, but Wu and Huang have updated the initialisation phase in^34^ from using $\mathcal {P}_{384}$ to $\mathcal {P}_{640}$. This change was made after a recent differential forgery attack due to^35^.
- DA2 uses all 96 bits of the nonce but observes the keystream after applying *r* rounds of permutation after the initialization phase.

In DA3 to DA5, the authors use the plaintext bits to build new distinguishers. Since, our focus is towards building distinguishers based on nonce bits only, we will be skipping DA3 to DA5 in further discussions.

In Table 2, we have given the best cubes mentioned in^36^ for DA2 where the authors have used cube indices from 64 to 95, i.e., only $nonce_2$. The zero-sum value is observed at the 64*th* index of the final state.

**Table 2:** Cube attacks from^36^.

Cube-size	Cube indices	Rounds
8	69, 70, 71, 76, 81, 86, 91, 92	419
14	64, 65, 70, 72, 76, 77, 78, 81, 85, 86, 87, 88, 92, 95	435
18	66, 67, 68, 72, 73, 75, 77, 79, 81, 82, 83, 84, 87, 88, 89, 90, 93, 94	437
18	67, 68, 69, 70, 72, 73, 75, 79, 80, 81, 83, 84, 85, 88, 89, 90, 91, 95	438

In our experiments, we will consider distinguishing attacks (as well as key-recovery in the next section) for the keyed permutation $\mathcal {P}_n$. The experiment is as follows.

1. We start with a state with all the bits set to 0.
2. The bits that are accessible to the attacker are $\{96, 97, \dots , 127\}$, i.e., the nonce bits. (Refer to Algorithm 2 ).
3. After the public bits are set, the state is updated using $\mathcal {P}_n$.
4. Finally, the attacker is only given access to the bits at positions $\{64, 65, \dots , 96\}$.

The designers of $\text{ TinyJAMBU }$^34^ claimed the following (directly quoting from^34^[p. 28]):

*We consider the algebraic property for the input bits at* $s_{96}\cdots s_{127}$. *Our experiment shows that after* 512 *rounds, every output bit at* $s_{64}\cdots s_{95}$ *is affected by the 32-bit input cube tester at* $s_{96}\cdots s_{127}$.

So, building distinguishers using 32-sized cubes is not possible.

We use a 7 size cube $\{69, 70, 76, 81, 86, 91, 92\}$. In our simulations, we use a linear congruential algorithm and 48-bit integer arithmetic in C to generate random binary values. We use 1 million random keys to find the bias. We get $\Pr (\text{ Superpoly }=1)=0.285 ~(0.485)$ for 434 (456) rounds.

**Figure 2:**
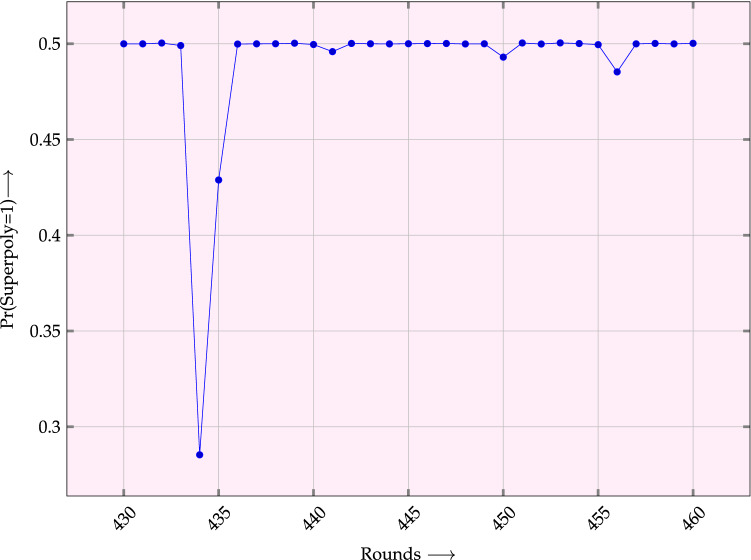
Probability of superpoly being 1 for different rounds for the cube $\{69, 70, 76, 81, 86, 91, 92\}$.

We present our experimental results in Fig. 2.

### How to find good cubes

**Figure 3:**
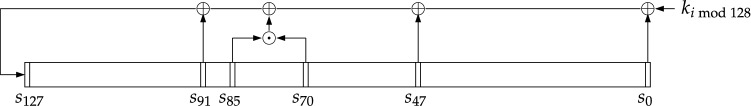
Feedback polynomial in $\mathcal {P}$.

The state bits of $\text{ TinyJAMBU }$ are updated using the nonce, associated data and plaintext using the permutation $\mathcal {P}$. Let us recall the feedback polynomial in permutation $\mathcal {P}$. Please see Fig. 3. It is a quadratic polynomial involving 5 state variables and 1 key variable. The only quadratic term in the polynomial is $s_{70}\cdot s_{85}$.

For the (weak-key) distinguishing attack, we will start with a state *s* with all 0 entries. As mentioned in “[Sec Sec6]” section, the 32 bit nonce is $\mathsf {XOR}$ed at the 96*th* position of the state. In any distinguishing attack, the desired cube must have two *good* properties.

1. The size of the cube must be small.
2. The number of rounds it can be used as a distinguisher must be large.

To achieve these properties simultaneously, one way is to make sure that the degrees of the polynomials in the state are as small as possible for the initial rounds. Observe that the the degree of the polynomials in the state can increase only due to the quadratic term in the permutation $\mathcal {P}$ function. Therefore, if we can control the quadratic term in the initial rounds, the degree of the polynomial can be minimized.

Since, the *feedback* polynomial has a non-linear term $s_{70} \cdot s_{85}$ (i.e., the terms are 15 indices apart) we just need to ensure that in the initial rounds, we can *minimize* the number of index pairs $(i, i+15)$ such that at most one among $s_i$ or $s_{i+15}$ contains a nonce variable. In doing so, the *feedback* polynomial will become linear for up-to certain rounds. Therefore, if we set a cube $\mathcal {C} = \{c_i\ |\ 0 \le i < 32\}$ such that $(c_i, c_{i+15}) \not \subseteq \mathcal {C}, \forall i \in \{0, 1, \dots , 16\}$, then we can ensure for larger number of rounds, the state will contain linear polynomials only.

For example, suppose we consider two cube $\mathcal {C}$ and $\mathcal {C'}$ such that $(c_0, c_{15}) \subseteq \mathcal {C}$ whereas $c_0 \in \mathcal {C'}, c_{15} \notin \mathcal {C'}$. As mentioned earlier, we will start with a state with all 0 bits and the 32-bit nonce is $\mathsf {XOR}$ed at index 96*th*. This implies that at round 0, we have $s_{96} = c_0$ and $s_{111} = c_{15}$ while considering the cube $\mathcal {C}$. Also, $s_i = 0$ for all $0 \le i < 96$. After 26 rounds, the state would get updated in such a way that $s_{70} = c_0$ and $s_{85} = c_{15}$. Observe that all the polynomials of the state are linear upto this round. But, in the 27*th* round, the feedback polynomial will contain the term $c_0 \cdot c_{15}$ and $s_{127}$ will become quadratic. When we consider the cube $\mathcal {C'}$, then after 26 rounds, we will get $s_{70} = c_0$ and $s_{85}$ will be some constant. Therefore, $s_{127}$ in the 27*th* will also be *linear*.

### Getting cubes for higher rounds

The best distinguisher mentioned in^36^ works for 438 rounds and uses a cube of size 18. In this subsection, we will present weak-keys for $\text{ TinyJAMBU }$ where the number of rounds is greater than 450. To begin with, we will use the cubes mentioned in “How to find good cubes” section which gives us an advantage over other cubes by making the feedback polynomials linear for more rounds.

After picking a good cube, we will follow the steps mentioned in “Weak-keys attacks using cubes from^36^ section to find key constraints so that we can build better distinguishers, i.e., we will carefully analyse the feedback polynomial at each round and try to set up constraints on the key variables so that either we are able to decrease the degree of the polynomial with respect to nonce variable or completely eliminate a term from the polynomial.

For example, let us consider the 14 sized cube $\mathcal {C}_I$ where

$$\begin{aligned} I = \{65, 66, 67, 68, 69, 70, 71, 72, 73, 74, 75, 76, 77, 78\} \end{aligned}$$

The rest of the nonce variables are set to 0. Observe that the set *I* has the required property that ensure that $\mathcal {C}_I$ is a good cube, i.e., $(i, i+15) \not \subseteq I$. At the 59*th* rounds, the feedback polynomial is

5 $$\begin{aligned} k_0\cdot k_{15} + k_0\cdot x_{10} + k_0 + k_{15} + k_{21} + k_{58} + x_9 + x_{10} + 1\,. \end{aligned}$$

By setting $k_0 = 1$, the polynomial gets reduced to

6 $$\begin{aligned} k_{21} + k_{58} + x_9 + 1\;. \end{aligned}$$

Observe that even though the degree of the polynomial with respect to nonce variable has not decreased, but the nonce variable $x_{10}$ does not appear in the polynomial. This allows us to control the mixing of nonce variables in subsequent rounds. Similarly, we set $k_1 = k_2 = k_3 = k_4 = 1$ for the next four rounds.

At the 65*th* round, the feedback polynomial is

7 $$\begin{aligned} k_6\cdot k_{21} + k_6 + k_{21}\cdot x_1 + k_{21} + k_{27} + k_{64} + x_1 + 1\;. \end{aligned}$$

In this case, if we set $k_{21} = 1$, the polynomial gets reduced to $k_{27}+k_{64}$, i.e., the degree of the polynomial with respect to nonce variable gets reduced to 0 as well as the number of monomials. By analysing the feedback polynomial in the next 13 rounds, we set $k_i = 1, \forall i \in \{22, 23, \dots , 34\}$.

Let us now focus on the 94*th* round. Here, we have

8 $$\begin{aligned} k_{12} + k_{13}\cdot k_{35} + k_{13} + k_{19} + k_{35}\cdot k_{50} + k_{35}\cdot x_{1} + k_{35}\cdot x_{8} + k_{50} + k_{56} + k_{93} + x_{1} + x_{8} + x_{14}\;. \end{aligned}$$

We set $k_{35} = 1$ to reduce the feedback polynomial. The same goes for $k_{i}, \forall i \in \{35,36, \dots ,$ $42\}$. By analysing a few rounds after the $102{nd}$ round, we set $k_{i} = 1, \forall i \in \{58, 59, 60, 61, 62\}$. Giving an addition 10 key constraints $k_i = 1, \forall i \in \{64, \dots , 73\}$, we get a zero sum distinguisher for 451 rounds. Thus, we have a total of 41 constraints on key for this case.

Next, we relax a few conditions on the key and check experimentally whether we are getting bias or not. We check that if we take $k_i=1 \ \forall i \in \{ 3, 4, 24, 26, 28, 29, 33, 34, 35, 40, 41, 71, 72\}$, still we get zero sum distinguisher for 451 rounds. Thus instead of 41 constraints, we now need only 13 constraints.

**Table 3:** Weak-key attacks for more than 450 rounds. Constraints are $k_i=1$ for $i \in \mathcal {I}$. Fourth column represents the probability of the superpoly being 1.

Cube indices	$\mathcal {I}$	Rounds	Prob.
65, 66, 67, 68, 69, 70, 71, 72, 73, 74, 75, 76, 77, 78	{3, 4, 24, 26, 28, 29, 33, 34, 35, 40, 41, 71, 72}	451	0
64, 65, 66, 68, 69, 70, 71, 72, 73, 74, 75, 76, 77, 78	{0, 1, 2, 3, 4, 21, 23, 24, 28, 29, 30, 32, 36, 37, 38, 39, 40, 42}	455	0.476
64, 65, 66, 67, 68, 70, 71, 72, 73, 74, 75, 76, 77, 78	{ 2, 3, 20, 21, 22, 25, 26, 27, 28, 32, 33, 34, 35, 36, 37, 38, 39, 40, 41, 42, 57, 58, 59, 61, 64, 66, 70, 71, 72, 73, 74, 76, 77}	466	0.467
81, 82, 83, 84, 85, 86, 87, 88, 89, 90, 91, 93, 94, 95	{0, 1, 6, 7, 8, 13, 14, 15, 16, 17, 20, 37, 38, 41, 44, 45, 50, 51, 52, 57}	476	0.478

In Table 3, we present our experimental results. We use $10^5$ random keys to find the probabilities. All cubes are of size 14. One can see from the first row that superpoly is always equal to 0 for 451 rounds if key satisfies 13 constraints. From the last row, it is clear that one can distinguish $\text{ TinyJAMBU }$ for 476 rounds if the secret key satisfies 20 constraints. Thus size of the corresponding weak-key class is $2^{108}$. Without any constraint, we get an approximate probability 0.5 in all the four cases.

## Exact degree in nonce of the feedback polynomial in $\mathcal {P}_n$

In this section, we will use the concept of monomial trails to find the exact degrees in nonce of the feedback polynomials for some rounds of $\text{ TinyJAMBU }$. To find the exact degrees, we will use the algorithms mentioned in^37^ and discuss the important changes required to adapt those algorithms into our scenario.

The exact degree of a polynomial $f(\textbf {x})$ can be written as $deg(f) = \underset{\pi _{\textbf {u}}(\textbf {x}) \rightarrow f(\textbf {x})}{\max } (\mathsf {wt}(\textbf {u}))$. Using Claim 2, we have a procedure to find the exact degree of $f(\textbf {x})$ given as follows.

1. Find a highest degree monomial $\pi _{\textbf {u}}(\textbf {x})$ such that $\pi _{\textbf {u}}(\textbf {x})  {\leadsto }f(\textbf {x})$.
2. If $|\pi _{\textbf {u}}(\textbf {x})  {\leadsto }f(\textbf {x})|$ is odd, then return $\mathsf {wt}(\textbf {u})$. Else, repeat the whole process until we find a new monomial whose degree is the largest and has an odd number of monomial trails.

We will use Mixed Integer Linear Programming (MILP) to find monomial trails and use Gurobi to solve the MILP instance. The objective function is to maximize the weight of $\textbf {u}$ whereas the constraints will be set up in such that a solution $\textbf {u}$ will ensure a monomial trail $\pi _{\textbf {u}}(\textbf {x})  {\leadsto }f(\textbf {x})$. To count the number of monomial trails, we will use *PoolSearchMode* in Gurobi which will help us to find multiple solutions. Here, we are using the fact that each solution of the model is a monomial trail $\pi _{\textbf {u}}(\textbf {x})  {\leadsto }f(\textbf {x})$.

**Figure 4:**
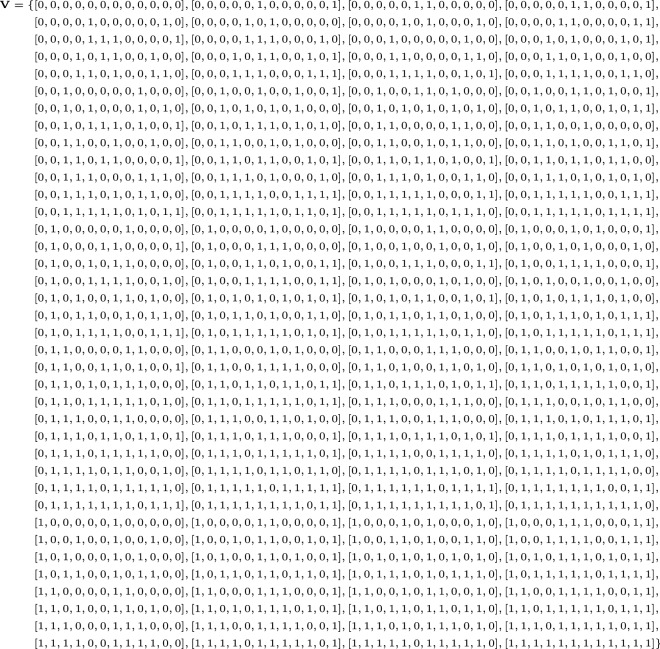
Set $$\textbf {V}$$ .

**Figure 5:**
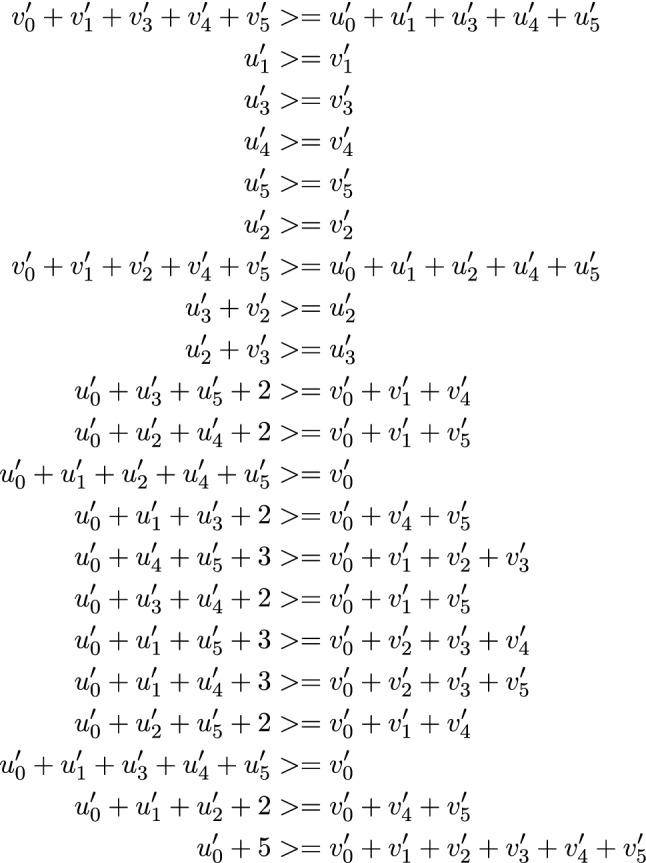
Inequalities for $$\mathcal {F}$$.

We now focus on the MILP model for finding the exact degree in nonce of the feedback polynomial of $\mathcal {P}_n$. To make the concepts easy to understand, we will consider the permutation $\mathcal {P}$ taking as input the state $\textbf {s}$ and key $\textbf {k}$ and output an updated state $\textbf {s}'$ but the same key $\textbf {k}$. Observe that the keyed permutation $\mathcal {P}_n$ can be written as

9 $$\begin{aligned} \mathcal {P}_n = \underbrace{\mathcal {P}\circ \mathcal {P}\circ \dots \circ \mathcal {P}}_{n \ times} \end{aligned}$$

and the feedback polynomial at the *n* *th* round is $\pi _{\textbf {e}_{128}}(\mathcal {P}_n(\textbf {s},\textbf {k}))$ where

10 $$\begin{aligned} \textbf {e}_{128} = [0,0, \dots , 0, \underset{128{th}\ index}{1}, 0, \dots , 0,0] \end{aligned}$$

From the definition of monomial trail, a trail exists from $\pi _{\textbf {u}^{(0)}}(\textbf {s})$ to $\pi _{\textbf {e}_{128}}(\mathcal {P}_n(\textbf {s},\textbf {k}))$ (denoted as $\pi _{\textbf {u}^{(0)}}(\textbf {s})  {\leadsto }\pi _{\textbf {e}_{128}}(\mathcal {P}_n(\textbf {s},\textbf {k}))$) if there is a set of binary vectors $\textbf {u}^{(1)}, \textbf {u}^{(2)}, \dots , \textbf {u}^{(n-1)}$ such that

11 $$\begin{aligned} \pi _{\textbf {u}^{(i-1)}}(\mathcal {P}_{i-1}(\textbf {s},\textbf {k})) \rightarrow \pi _{\textbf {u}^{(i)}} (\mathcal {P}_i(\textbf {s},\textbf {k})), \forall i \in [128] \end{aligned}$$

where $\textbf {u}^{(128)} = \textbf {e}_{128}$ and $\textbf {s}$ is the initial state such that $s_{96 + i} = x_i, \forall i \in \{0, 1, \dots , 32\}$ and the rest are 0. Therefore, we only need to find out linear constraints consisting of $\textbf {u}^{i-1}$ and $\textbf {u}^{i}$ such that the constraints are satisfied if and only if $\pi _{\textbf {u}^{(i-1)}}(\mathcal {P}_{i-1}(\textbf {s},\textbf {k})) \rightarrow \pi _{\textbf {u}^{(i)}} (\mathcal {P}_i(\textbf {s},\textbf {k}))$. For the sake of simplicity, let us consider the notation $(\textbf {y}, \textbf {k}) := \pi _{\textbf {u}^{(i-1)}}(\mathcal {P}_{i-1}(\textbf {s},\textbf {k}))$, then $(\textbf {z}, \textbf {k}) :=\pi _{\textbf {u}^{(i)}} (\mathcal {P}_i(\textbf {s},\textbf {k})) = \mathcal {P}(\textbf {y},\textbf {k})$. Also, we will use $\textbf {u}:= \textbf {u}_{(i-1)}$ and $\textbf {v}:= \textbf {u}^{(i)}$. So, we need to find linear constraints $\textbf {u}$ and $\textbf {v}$ such that the constraints are satisfied if and only if $\pi _{\textbf {u}}(\textbf {y},\textbf {k}) \rightarrow \pi _{\textbf {v}}(\textbf {z},\textbf {k})$.

Observe that from the description of $\mathcal {P}$, we have $z_i = y_{i+1}, \forall i \in \{0,1,\dots ,126\}$ and $z_{127}$ depends on five entries of *y* and a single key entry. In other words, only one entry in *z* is different from *y* and it depends only on few entries of the input. Let us capture this in a function $\mathcal {F}$ which takes as input $(y_0, y_{47}, y_{70}, y_{85}, y_{91}, k_i)$ and outputs $(z_{127}, z_{46}, z_{69}, z_{84}, z_{90}, k_i)$ such that

12 $$\begin{aligned} z_{i+1}= & {} y_{i}, \forall i \in \{47, 70, 85, 91\} \end{aligned}$$

13 $$\begin{aligned} z_{127}= & {} y_0 + y_{47} + y_{70}\cdot y_{85} + y_{91} + k_i \end{aligned}$$

Apart from $(z_{127}, z_{46}, z_{69}, z_{84}, z_{90}, k_i)$, the rest of the $z_j$’s and $k_j$’s are equal to some $y_j$’s or $k_j$’s or they are not involved in the function $\mathcal {F}$. Therefore, in the MILP model, we can set those $z_j$’s and $k_j$’s to their corresponding $y_j$’s or $k_j$’s. We only need to figure out constraints on $\textbf {u}'$ and $\textbf {v}'$ such that $\prod _{\textbf {u}'}(y_0, y_{47}, y_{70}, y_{85}, y_{91}, k_i) \rightarrow \prod _{\textbf {v}'}(z_{127}, z_{46}, z_{69}, z_{84}, z_{90}, k_i)$.

We begin by enumerating all binary vectors of dimension 6 into a set $\textbf {V}$ (see Fig. 4) such that $[\textbf {u}', \textbf {v}'] \in \textbf {V}\iff \pi _{\textbf {u}'}(y_0, y_{47}, y_{70}, y_{85}, y_{91}, k_i) \rightarrow \pi _{\textbf {v}'}(z_{127}, z_{46}, z_{69}, z_{84}, z_{90}, k_i)$. Using the set $\textbf {V}$, we can generate the linear constraints that define the convex hull of $\textbf {V}$. This can be achieved by using the *inequality_generator()* function in SAGE^41^. Observe that the convex hull generated does not contain any spurious points because the points in $\textbf {V}$ are on the surface of a 12 dimensional unit cube. Therefore, the convex hull is bounded by the unit cube unless the hull is unbounded, which is not the case. Since, the interior of the unit cube does not contain any integer points, the convex hull won’t contain any spurious points.

Since, the number of inequalities affects the running time of the MILP solver, one needs to make sure they work with minimal number of constraints. For this, we go through the inequalities generated through SAGE and check one by one whether any inequality can be removed. By doing so, we arrive at a system of 27 linear inequalities that describes $\pi _{\textbf {u}'}(y_0, y_{47}, y_{70}, y_{85}, y_{91}, k_i) \rightarrow \pi _{\textbf {v}'}(z_{127}, z_{46}, z_{69}, z_{84}, z_{90}, k_i)$ as shown in Fig. 5.

Now we have the required inequalities (which we will denote an $\mathcal {L}$), we will describe the MILP model for finding largest monomial that has a trail to the feedback polynomial of $\mathcal {P}_n$. The objective function is to maximize $\sum _{i = 96}^{127} u_i^{(0)}$. This is because the nonce variables are present at those index. We must also set $u_i^{(0)} == 0, \forall i < 96$ as we are not interested in monomials containing $s_i$ where $i < 96$. Using the set $\mathcal {L}$, we will create linear inequalities for $\textbf {u}^{(i-1)}$ and $\textbf {u}^{(i)}$ which will ensure that $\pi _{\textbf {u}^{(i-1)}}(\mathcal {P}_{i-1}(s,k)) \rightarrow \pi _{\textbf {u}^{(i)}} (\mathcal {P}_i(s,k))$. Finally, since we are interested in the feedback polynomial of $\mathcal {P}_n$, we will set $u_{127}^{(n)} == 1$ and the rest to 0.

**Figure 6:**
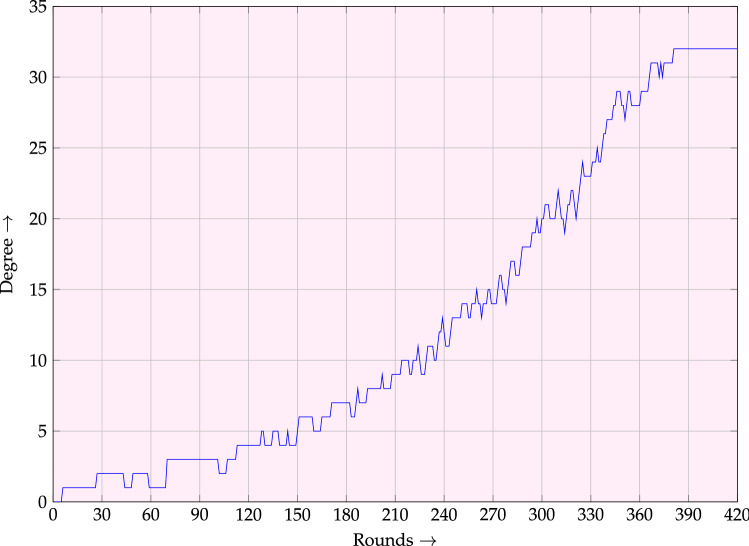
Degree of feedback polynomial .

Solving the above model using Gurobi^42^, will only give us information about the largest term that has a monomial trail. To conclude that the term is also a monomial in the feedback polynomial, we need to count the number of monomial trails. Observe that an optimal solution in the above model is a valid monomial trail, i.e., $\textbf {u}^{(i)}$’s gives us all the information of the trail. Therefore, it is enough to count the number of solutions in the model by only fixing $\textbf {u}^{(0)}$ to the one returned by optimal. The number of solutions can be obtained by using the *PoolSearchMode* of Gurobi which searches for all the solutions. One can see from Fig. 6 that the degree of the feedback polynomial after 381 rounds is 32.

## Key-recovery attacks

In this section, we will present key-recovery attacks against $\text{ TinyJAMBU }$. Like other key-recovery attacks, our attacks consist of two phases: offline and online phases. In the offline phase, the attacker finds polynomials consisting of key variables by using methods such as cube attacks, MILP, etc. Once the attacker is equipped with these polynomials, in the offline phase, he will try to obtain the evaluations of these polynomials and recover certain key bits.

### Offline phase: finding the polynomials

In the offline phase of our attack, we will use MILP to extract the polynomials with key variables. Let $\mathcal {C}_I$ be a cube with index set *I* and the output polynomial written with respect to this cube be

14 $$\begin{aligned} f(\textbf {x}, \textbf {k})\;=\; \textbf {t}\cdot p_{\textbf {t}}(\textbf {x}, \textbf {k}) + q_{\textbf {t}}(\textbf {x}, \textbf {k})\;, \end{aligned}$$

where $\textbf {t}= \prod _{i \in I} x_i$. Let $\textbf {A}= \{a_i \ |\ i \notin I\}$ be the set of constants for the non-cube variables. Our objective is to find the superpoly $p_{\textbf {t}}(A, \textbf {k})$ which will be used in the online phase. We will again use the concept of monomial trails to find the superpolies. This technique was also used in^37^ to find the superpolies for Trivium.

In our attack, we will consider $\textbf {A}$ to be all zeroes. Recall that in “[Sec Sec14] section, we presented a MILP model that would find the degree of the largest monomial containing nonce variables only that has a monomial trail to the feedback polynomial. This is achieved by setting up a system of linear inequalities with the objective function being “maximize $\sum _{i=96}^{127} u_i^{(0)}$”. Then, by counting the number of monomial trails, we can confirm whether it is monomial in the feedback polynomial or not. We will simply modify the above MILP model that will help us find every monomial and build the entire superpoly.

We first begin by modifying the MILP model. The objective function must be changed to maximizing $\sum _{i=128}^{255} u_i^{(0)}$. Recall that we considered the input to $\mathcal {P}$ as $(\textbf {s},\textbf {k})$ were $\textbf {s}$ is the state and $\textbf {k}$ is the key. Therefore, the objective function is looking for the largest term consisting of key variables. Since, we are interested in the superpoly with respect to some $\textbf {t}$, we need to add the following equalities as constraints also.

15 $$\begin{aligned} u_i^{(0)} == 1,&\forall i \in \textbf {I}\end{aligned}$$

16 $$\begin{aligned} u_i^{(0)} == 0,&\forall i \in \{0,1,\dots ,127\} \setminus \textbf {I}\end{aligned}$$

The rest of the inequalities are exactly same as the ones used in “[Sec Sec14] section. Observe that a solution to the above MILP model gives us a monomial $\textbf {x}_{\textbf {I}} \textbf {k}_{\textbf {J}}$ for some index set $\textbf {J}$ such that $|\textbf {J}|$ is the largest and $\textbf {x}_{\textbf {I}} \textbf {k}_{\textbf {J}}$ has a monomial trail to $\mathcal {P}_n(\textbf {s},\textbf {k})^{(127)}$. To confirm that this is a monomial, we must again count the number of solutions and store it if it is a monomial. Now, to find the next monomial, we must add a new constraint so that the MILP solver won’t return the same solution again. To be precise, suppose the MILP solver returns a solution $\textbf {u}^{(0)}$. For the sake of simplicity, let $\textbf {J} = \{i \ |\ u_i^{0} = 1, i \ge 128\}$. Then, we add the constraint,

17 $$\begin{aligned} \sum _{i \in J} (1-\textbf {u}_i^{(0)}) >= 0 \end{aligned}$$

This constraint ensures that the MILP solver won’t return the same solution twice.

In Table 4, we present a few superpolies of the feedback polynomial with respect to cubes of size 31 for $\mathcal {P}_{376}$ by solving the MILP model mentioned above.

**Table 4:** Superpolies for $\mathcal {P}_{376}$.

Non-cube indices	Superpoly
4	$k_{13} + k_{14} + k_{20}$
5	$$k_{14} k_{28}+k_{14} k_{22}+k_{7} k_{14}+k_{14} k_{20}+k_{14} k_{65}+k_2 k_{14}$$ $$+k_{13} k_{14}+k_2 k_{22}+k_{14} k_{42}+k_2 k_{72}+k_2 k_6+k_7 k_{20}+k_{44} k_{57}+k_{2} k_7$$ $$+k_1 k_{57}+k_1 k_{13}+k_{20} k_{22}+k_2 k_{87}+k_2 k_{50}+k_{50} k_{72}+k_{20} k_{28}+k_{20} k_{65}+k_{13} k_{44}+k_{13} k_{65}$$ $$+k_{13} k_{87}+k_{14} k_{45}+k_{35} k_{72}+k_1 k_{45}+k_{2} k_{28}+k_2 k_{65}+k_{50} k_{87}+k_{13} k_{72}+k_{6} k_{72}$$ $$+k_{57} k_{65}+k_{14} k_{34}+k_{44} k_{45}+k_{35} k_{87}+k_6 k_{87}+k_{35} k_{45}+k_{45} k_{65}+k_2+k_{87}$$ $$+k_{72}+k_{20}+k_{78}+k_{34}+k_{42}+k_{65}+k_{57}+k_{71} $$
6	$$k_{4} k_{7} k_{14} + k_{1} k_{22} + k_{22} k_{44} + k_{2} k_{14} $$ $$+ k_{4} k_{7} + k_{2} k_{44} + k_{7} k_{22} + k_{1} k_{2} + k_{7} k_{22} + k_{2} k_{44} + k_{1} k_{22} $$ $$+ k_{22} k_{44} + k_{4} k_{7} + k_{1} + k_{44} + k_{22} + k_{28}$$
14	$k_{7}k_{14} + k_{7}$
13	$k_{4} k_{7} k_{14} + k_{4} k_{14} + k_{4} k_{7} + k_{7} k_{22} + k_{1} k_{22} + k_{22} k_{4} + k_{14} + k_{22} + k_4 + k_{28} + k_{65}$
16	$k_{2} k_{7} k_{14} + k_{7} k_{12} k_{14} + k_{7} k_{14} + k_{6} k_{14} + k_{14} k_{50} + k_{7} k_{35} + k_{14} + k_{50} + k_{7} + k_{6} + k_{35}$
17	$k_{4} k_{14} + k_{4} + 1$
20	$k_1 + k_{14} + k_{44} + k_{65}$
28	$k_{14} + k_{72} + k_{56} + k_{12} + k_{35} + k_{50} + k_{87} + k_{34} + k_{19} + k_{93}$
29	$k_{1} + k_{14} + k_{44}$
31	$k_1 + k_2 + k_{44} + k_{45}$

### Online phase: recovering the key bits

In the online phase, the attacker is given a blackbox access to $\text{ TinyJAMBU }$, i.e., the attacker is allowed to choose the value of 32-bit nonce and is given the bits at position $64, 65, \dots , 95$ of the state updated by $\mathcal {P}_n$. Observe that there are 5 linearly independent polynomials in Table 4. This implies that the attacker can get the evaluations of these superpolies by performing cube tester for 440 round $\text{ TinyJAMBU }$. Therefore, the attacker can recover the entire key in time $2^{123} + 5\cdot 2^{31}$.

## Conclusion

In this work, we presented the *first* weak-key attack against $\text{ TinyJAMBU }$. We also study the exact degree in nonce of the feedback polynomial of keyed permutation $\mathcal {P}_n$ and give better key recovered attack for $\text{ TinyJAMBU }$. As future work, we would like to investigate the following questions.

1. Similar to the DA3 attack considered in^36^, can we give distinguisher for rounds greater than 480 by using all 96 bits of the nonce?
2. Is there a practical key recovery attack for $\text{ TinyJAMBU }$ where the number of rounds is more than 450?
3. Does there exist any other inherent weakness for the permutation $\mathcal {P}_n$ which can be exploited to get better cubes or used in any attacks?

## Data Availability

One can generate data using our source codes. The source codes of our results are publicly available at https://drive.google.com/file/d/1nkyJiogxueZUg5gQvESBkAtg6WKHum1p/view.
